# Layer chicken microbiota: a comprehensive analysis of spatial and temporal dynamics across all major gut sections

**DOI:** 10.1186/s40104-023-00979-1

**Published:** 2024-02-05

**Authors:** Yadav Sharma Bajagai, Thi Thu Hao Van, Nitish Joat, Kapil Chousalkar, Robert J. Moore, Dragana Stanley

**Affiliations:** 1 Institute for Future Farming Systems, Central Queensland University, Rockhampton, QLD 4701 Australia; 2 School of Science, RMIT University, Bundoora, VIC 3083 Australia; 3 School of Animal and Veterinary Sciences, The University of Adelaide, Roseworthy, South Australia 5371 Australia

**Keywords:** Chicken microbiota, Gut microbiota, Layers, Spatial variation, Temporal variation

## Abstract

**Background:**

The gut microbiota influences chicken health, welfare, and productivity. A diverse and balanced microbiota has been associated with improved growth, efficient feed utilisation, a well-developed immune system, disease resistance, and stress tolerance in chickens. Previous studies on chicken gut microbiota have predominantly focused on broiler chickens and have usually been limited to one or two sections of the digestive system, under controlled research environments, and often sampled at a single time point. To extend these studies, this investigation examined the microbiota of commercially raised layer chickens across all major gut sections of the digestive system and with regular sampling from rearing to the end of production at 80 weeks. The aim was to build a detailed picture of microbiota development across the entire digestive system of layer chickens and study spatial and temporal dynamics.

**Results:**

The taxonomic composition of gut microbiota differed significantly between birds in the rearing and production stages, indicating a shift after laying onset. Similar microbiota compositions were observed between proventriculus and gizzard, as well as between jejunum and ileum, likely due to their anatomical proximity. *Lactobacillus* dominated the upper gut in pullets and the lower gut in older birds. The oesophagus had a high proportion of Proteobacteria, including opportunistic pathogens such as *Gallibacterium*. Relative abundance of *Gallibacterium* increased after peak production in multiple gut sections. *Aeriscardovia* was enriched in the late-lay phase compared to younger birds in multiple gut sections. Age influenced microbial richness and diversity in different organs. The upper gut showed decreased diversity over time, possibly influenced by dietary changes, while the lower gut, specifically cecum and colon, displayed increased richness as birds matured. However, age-related changes were inconsistent across all organs, suggesting the influence of organ-specific factors in microbiota maturation.

**Conclusion:**

Addressing a gap in previous research, this study explored the microbiota across all major gut sections and tracked their dynamics from rearing to the end of the production cycle in commercially raised layer chickens. This study provides a comprehensive understanding of microbiota structure and development which help to develop targeted strategies to optimise gut health and overall productivity in poultry production.

**Supplementary Information:**

The online version contains supplementary material available at 10.1186/s40104-023-00979-1.

## Background

The importance of gut microbiota in the health, welfare and productivity of chicken is well established. The gut microbiota influences various aspects of host physiology, metabolism, and immune function [1]. An important role of the intestinal microbiota is the fermentation or breakdown of complex polysaccharides present in plant cell walls to release short-chain fatty acids (SCFAs), such as acetate, propionate, and butyrate. These SCFAs serve as a valuable energy source for chickens and have several beneficial effects on the host [2]. In addition, the microbiota can contribute to the production of essential nutrients like amino acids and vitamins required by the host [3], assists in host immune system development and intestinal homeostasis [4, 5] and provides protection against pathogens [6]. Moreover, a balanced and diverse microbiota is associated with improved growth performance, efficient feed utilisation, reduced disease outbreaks, and better stress tolerance in chickens [1, 7, 8].

Understanding the complex relationship between gut microbiota and the host is key to developing strategies to improve productivity and health. Characterisation of the composition of the normal healthy gut microbiota is essential to understand how to nurture the microbiota to enhance overall well-being and performance.

Over the last decades many studies have investigated the gut microbiota of chickens, however, there are some limitations in the previous studies about chicken gut microbiota. First, most of the studies were on broiler chickens and thus only covered the relatively shorter lifespan of broilers. Also, the majority of the previous studies described the microbiota of only one or two sections of the intestine (mostly cecum, some with cecum and ileum), but there are very few studies which described the microbiota profile of all sections of the digestive system. Moreover, almost all the studies were conducted in controlled research environments, rather than in typical production settings. Finally, most microbiota studies collected samples at a single time point and thus failed to capture the temporal variation in microbiota structure. Laying hens have some distinct characteristics compared to broilers, such as genetic background, lifespan, and feeding and housing practices. Therefore, it is expected that these differences result in significant differences in their microbiota population. In this study, we have addressed all these limitations to study the microbiota throughout the gut of layer chickens in a commercial setting, over their complete productive lifespan.

In a natural setting, chickens have evolved to be hatched in a nest with an adult hen, and the microbiota of the hen and nest would provide a significant contribution to shaping the microbiota of the newly hatched chickens. Current industrial chicken production, in a clean hatchery setting without contact with mother hen, has disrupted this natural process of intestinal microbiota colonisation. This practice results in a variable and unpredictable microbiota composition because, microbiota structure in modern poultry production depends on the type of microorganisms present in the surrounding environment, feed, and water [9, 10]. Housing and production system may influence the microbiota structure. Therefore, we have collected samples from different production systems to capture this possible variation. Here, we present the spatial differences in microbiota structure in different sections of the digestive system with the temporal development of microbiota from rearing to later stages of production in commercial laying hens.

## Methods

### Study design and husbandry

Four commercial layer flocks—one each from free-range (Flock A) and barn housing (Flock B), and two from cage production systems (Flock C and Flock D)—were chosen for this study. The flocks were selected based on the farmers' willingness to participate in the study and their proximity to the laboratory. Flocks A and B were raised on dirt floors from hatching, before being moved to free-range and barn housing production systems. Flock C was raised on a concrete floor from hatch, and then moved to a multi-age cage production facility, while flock D was raised in cages from hatch to the later stages of production.

All four flocks were Hyline brown layers, that came from the same hatchery and were provided with commercial feed based on wheat and soybean manufactured by the same feed mill fulfilling the nutrient requirement recommended for the breed. All flocks were raised in separate farms during the same season. The birds were fed starter diet till week 6, grower diet from 7 to 12 weeks, developer diet from 13 to 15 weeks, pre-lay diet from 16 to 17 weeks and layer diet from week 18 onwards. The stocking density was 30 kg/m^2^ in flocks A, B and C, while stocking density was 27 kg/m^2^ in flock D. All four flocks were transferred from rearing facilities to production facilities at 16 weeks. Mortality during the rearing phase was less than 2% in all flocks.

All flocks were vaccinated against infectious bronchitis, infectious laryngotracheitis, Newcastle disease, avian encephalomyelitis, egg drop syndrome, and Marek's disease. Flock A was also vaccinated against coccidia and fowl pox in addition to the above vaccines. Flock A had an outbreak of Spotty Liver Disease (SLD) at the age of 34 weeks caused by *Campylobacter hepaticus* infection resulting in a 20% decline in egg production. The flock recovered in two weeks after receiving chlortetracycline treatment for one week.

This study was conducted in compliance with the standards stated in the eighth edition (2013) of the Australian Code for the Care and Use of Animals for Scientific Purposes, and the study was approved by the institutional Animal Ethics Committee of The University of Adelaide under the approval No. S-2018-015.

### Sample collection

Ten birds from each flock were randomly selected and euthanised by cervical dislocation at weeks 6, 10, 14, 18, 26, 40, 60 and 75. A total of 320 birds were euthanised for this study. A total of 2,880 samples (mucosal scrapping) were collected from the oesophagus, crop, proventriculus, gizzard, duodenum, jejunum, ileum, cecum, and colon and stored at −20 °C until DNA extraction.

### DNA isolation

The DNA from the samples was isolated and purified using the QIAamp Fast DNA Stool Mini kit (Qiagen) using a modified methodology [11] further optimised for this study. Briefly, 200 mg of samples were mixed with 390 mg of glass beads (180 mg of 450–600 µm and 210 mg of 106 µm diameter) and 1 mL of preheated (70 °C) InhibitEx buffer (Qiagen). The samples with beads and buffer were then vortexed and homogenised with a Bullet Blender (Next Advance) for 5 min and incubated on ice for 30 s and in a 95 °C heat block for 7 min. The samples were centrifuged for 2 min, and the supernatant was further processed with the modified QIAamp Fast DNA Stool Mini kit as described by Knudsen et al. [11]. Finally, the DNA was eluted with 100 µL of ATE buffer (Qiagen) and stored at −20 °C.

### 16S rRNA gene amplicon sequencing

The V3–V4 variable regions of the 16S rRNA gene were PCR amplified using dual indexing, variable spacer primer pair 338F (5´-ACTCCTACGGGAGGCAGCAG-3´) and 806R (5´-GGACTACHVGGGTWTCTAAT-3´) [12]. The PCR cycling parameters, using Q5 DNA polymerase (New England Biolabs), were 98 °C for 1 min, 35 cycles of 98 °C for 10 s, 49 °C for 30 s, and 72 °C for 30 s, with a final step of a 10-min extension at 72 °C. The amplicons were sequenced using Illumina MiSeq 2 × 300 bp paired-end technology.

### Bioinformatics and statistical analysis

The sequencing data were demultiplexed using Cutadapt [13], and the upstream analysis was conducted using Quantitative Insights into Microbial Ecology 2 (QIIME2) [14] with the DADA2 [15] plugin for quality filtering, denoising, and chimaera removal. The DADA2-identified amplicon sequence variant (ASV) features were further clustered with vsearch to Operational Taxonomic Units (OTUs) at 97% similarity and taxonomically classified using the SILVA v138.1 database [16].

The downstream statistical analysis and visualisation at OTU and higher levels of taxonomic classification were done within the R program using a range of packages, including Phyloseq [17], Phylosmith [18], Vegan [19], and Microeco [20], Microbiome [21], and MaAsLin2 [22]. The data were rarefied to 3,000 sequences per sample for all downstream analyses. The data were analysed globally and separately for each organ.

The birds were categorised into Pullets (week 6 to 14), LayOnset (week 18), PeakLay (week 26), MidLay (week 40 to 60) and LateLay (week 75) for analysis and visualisation purposes.

## Results

### Number of sequences and filtering

A total of ~115 million DNA sequences were obtained from 2,393 samples from various sections of the digestive system (oesophagus = 278, crop = 296, proventriculus = 275, gizzard = 292, duodenum = 246, jejunum = 223, ileum = 227, cecum = 274, and colon = 282). The number of sequences per sample ranged from 1,113 to 116,373. All the sequences with a frequency less than 0.001 were removed and then rarefied to 3,000 sequences per sample and samples with less than 3,000 sequences were excluded from the analysis. A total of 2,320 samples (oesophagus = 275, crop = 293, proventriculus = 272, gizzard = 278, duodenum = 229, jejunum = 206, ileum = 216, cecum = 272, and colon = 279) remained after filtering and rarefying and were used for all downstream analysis and visualisation.

### Overall microbiota profile and spatial variation

**Fig. 1:**
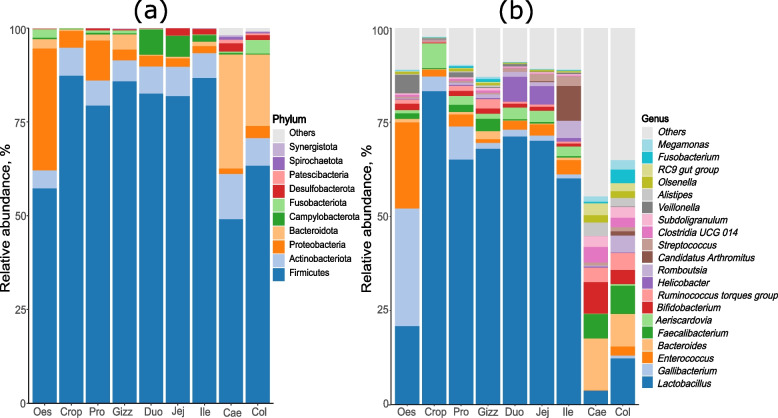
Phylum (**a**) and genus (**b**) level microbiota compositions in different intestinal sections of laying hens. Stacked bars represent the mean of all samples in the group. The phylum level plot shows the 10 most abundant phyla, and the genus level plot shows 20 most abundant genera. Oes = Oesophagus, Pro = Proventriculus, Gizz = Gizzard, Duo = Duodenum, Jej = Jejunum, Ile = Ileum, Cae = Caecum, Col = Colon

The top 10 bacterial phyla of the layer digestive system microbiota were Firmicutes, Actinobacteriota, Proteobacteria, Bacteroidota, Campylobacterota, Fusobacteriota, Desulfobacterota, Patescibacteria, Spirochaetota, and Synergistota (Fig. 1a, and Fig. S1). *Lactobacillus, Gallibacterium, Enterococcus, Bacteroides, Faecalibacterium, Aeriscardovia, Bifidobacterium, Ruminococcus torques group, Helicobacter, Romboutsia, Candidatus Arthromitus* (reclassified as *Candidatus* *Savagella* [23])*, Streptococcus, Clostridia UCG 014, Subdoligranulum, Veillonella, Alistipes, Olsenella, RC9 gut group, Fusobacterium,* and *Megamonas* were the top 20 most abundant genera (Fig. 1b and Fig. S2).

More than 90% of the microbiota in each organ was represented by the top 3 to 4 phyla in the respective organ. Firmicutes represented more than 75% of the relative abundance in the crop, proventriculus, gizzard, duodenum, jejunum, and ileum, while Firmicutes were more than 50% in the oesophagus and colon. The caecum was the only organ with less than 50% relative abundance of Firmicutes. The oesophagus had the highest proportion of Proteobacteria followed by the proventriculus and crop, while the caecum had the highest proportion of Bacteroidota followed by the colon. Actinobacteriota were represented in similar proportions in all organs, with the highest proportion in the caecum and the lowest in the oesophagus. The duodenum had the highest proportion of Campylobacterota followed by the jejunum and ileum, while the colon had the highest representation of Fusobacteriota followed by the oesophagus.

**Fig. 2:**
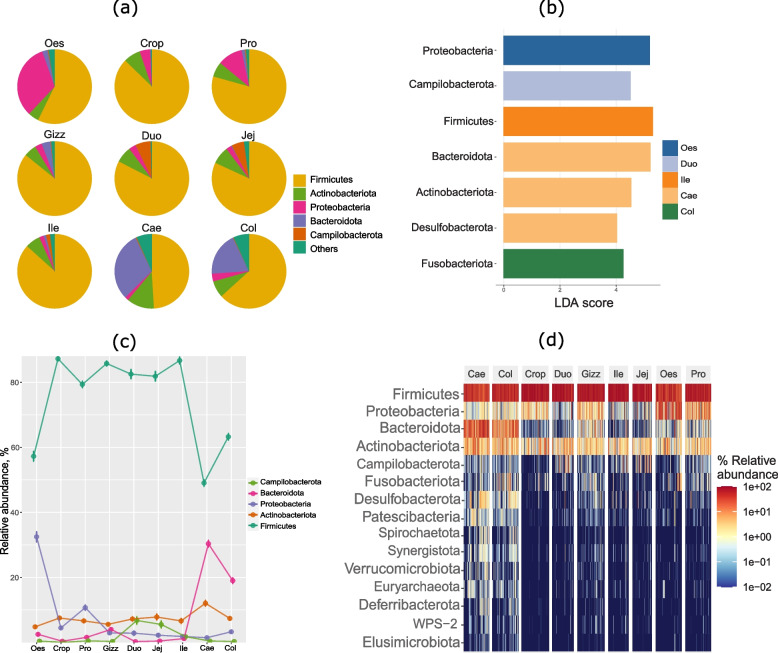
Microbiota composition at phylum level. (**a**) Pie chart showing the proportion of top 5 most abundant phyla in different sections of the gut. (**b**) Characteristic phyla of different sections as identified with linear discriminant analysis (LDA) effect size (LEfSe). Phyla with LDA scores of greater than 4 are presented. (**c**) Line graph depicting the relative abundance of top 5 most abundant phyla in different sections of the gut. (**d**) Heatmap showing the top 15 most abundant phyla in different sections of the gut. Oes = Oesophagus, Pro = Proventriculus, Gizz = Gizzard, Duo = Duodenum, Jej = Jejunum, Ile = Ileum, Cae = Caecum, Col = Colon

Although there were significant overlaps in the phylum level microbiota structure between organs, the linear discriminant analysis (LDA) effect size (LEfSe) biomarker discovery tool revealed that some of the phyla were differentially abundant and are indicators of the differences in microbiota between the various sections of the gastrointestinal tract. Proteobacteria was the defining phyla for the oesophagus, while the duodenum and ileum were defined by Campylobacterota and Firmicutes, respectively (Fig. 2b). Bacteroidata, Actinobacteriota, and Desulfobacterota were enriched in the caecum compared to other sections while the colon was characterised by higher relative abundance of Fusobacteriota.

*Lactobacillus* was by far the most dominant genus in the digestive system, with more than half of the microbiota represented by *Lactobacillus* in the crop, proventriculus, gizzard, duodenum, jejunum, and ileum. The caecum had the lowest proportion of *Lactobacillus*, followed by the colon and oesophagus (Fig. 1b).

**Fig. 3:**
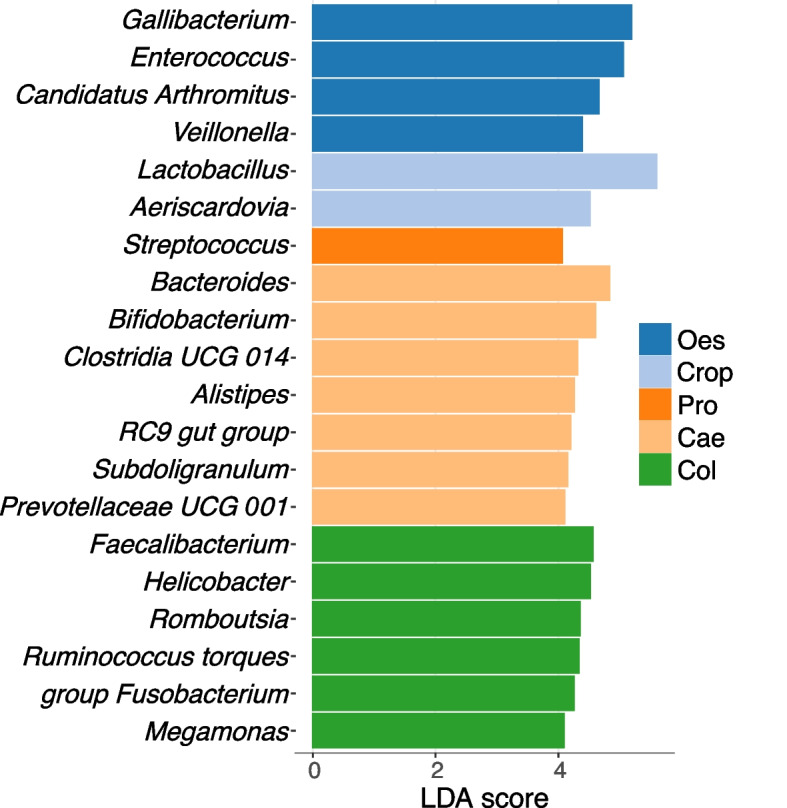
Differential genera in different sections of the gut. Differential genera were identified with the linear discriminant analysis (LDA) effect size (LEfSe) tool. Genera with LDA scores of greater than 4 are presented. Oes = Oesophagus, Pro = Proventriculus, Cae = Caecum, Col = Colon

LEfSe was used to identify the genera that most characterised each organ. *Gallibacterium, Enterococcus, Candidatus Arthromitus* (renamed as *Candidatus Savagella*) and *Veillonella* were the characterising genera of the oesophagus. *Lactobacillus* and *Aeriscardovia* characterised crop, while *Streptococcus* was the characterising genus for the proventriculus. The characterising genera for the caecum were *Bacteroides, Bifidobacterium, Clostridia UCG 014, Alistipes, RC9 gut group, Subdoligranulum,* and *Prevotellaceae UCG 001.* The colon was characterised by *Faecalibacterium, Helicobacter, Romboutsia, Ruminococcus torques group, Fusobacterium,* and *Megamonas* (Fig. 3). There were no bacterial taxa at the genus level differentially abundant in any of the small intestine locations (duodenum, jejunum and ileum).

**Fig. 4:**
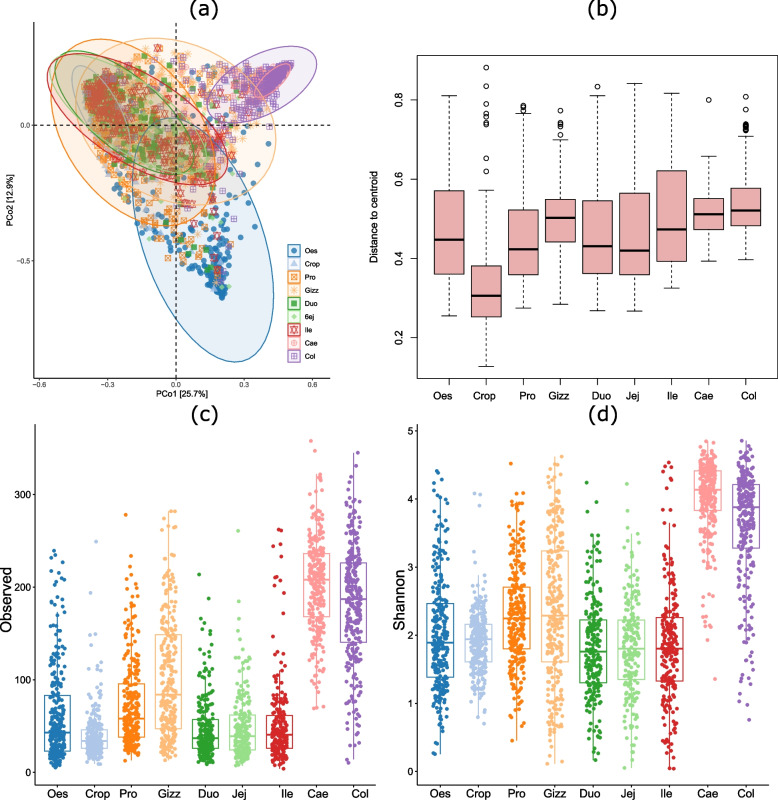
Diversity of microbiota in different sections of the gut. (**a**) Ordination of individual samples with principal coordinate analysis (PCoA) of Bray–Curtis distance. (**b**) Box plot showing the dispersion of samples in different gut sections measured as distance from centroid. (**c**) Microbiota richness in different gut sections measured with the indicator Observed taxa. (**d**) Microbiota alpha diversity in different gut sections as measured with the Shannon diversity index. Oes = Oesophagus, Pro = Proventriculus, Gizz = Gizzard, Duo = Duodenum, Jej = Jejunum, Ile = Ileum, Cae = Caecum, Col = Colon

The oesophagus, cecum, and colon had the most strikingly different microbiota populations. In contrast, the microbiota of other sections of the gut had significant overlap (Fig. 4a). The cecum, colon, and gizzard had the least sample-to-sample variation and the highest distance to the centroid. In contrast, other organs had relatively higher sample-to-sample variation and lower distance to the centroid (Fig. 4b).

### Spatial differences in microbiota richness and diversity

Cecum had the highest microbiota richness and diversity, followed by the colon, while crop, duodenum, jejunum, and ileum had the lowest richness and diversity (Fig. 4c and d). There were no significant differences in microbiota richness measured with the Observed species and microbiota diversity measured with the Shannon alpha diversity index among crop, duodenum, jejunum, and ileum (Dunn's Kruskal–Wallis Multiple Comparisons, *P* > 0.05). Although the oesophagus had a higher microbiota richness than that in the crop (*P* > 0.005), there were no differences (*P* > 0.05) in richness and diversity in the oesophagus compared to all small intestine sections (duodenum, jejunum, ileum). Gizzard had a higher microbiota richness (Observed species, *P* < 0.01) but similar diversity (Shannon index, *P* > 0.05) when compared to the proventriculus. Cecum had a similar richness (Observed species, *P* > 0.05) but higher diversity when compared with the colon (Shannon index, *P* < 0.01). Comparisons of all major richness and diversity indices in all organs are shown in the supplementary Table S1.

Figure 4a depicts the ordination of the samples with PCoA in different organs based on their beta diversity calculated with the Bray–Curtis dissimilarity index, whereas Fig. 4b shows the dispersion of the samples from the centroid. The cecum and colon had the least variability in the sample-to-sample distance, while the upper gut (oesophagus and crop) had higher variability in the sample-to-sample distance. All small intestinal section had comparable variability in the sample-to-sample distance.

### Temporal variation

**Fig. 5:**
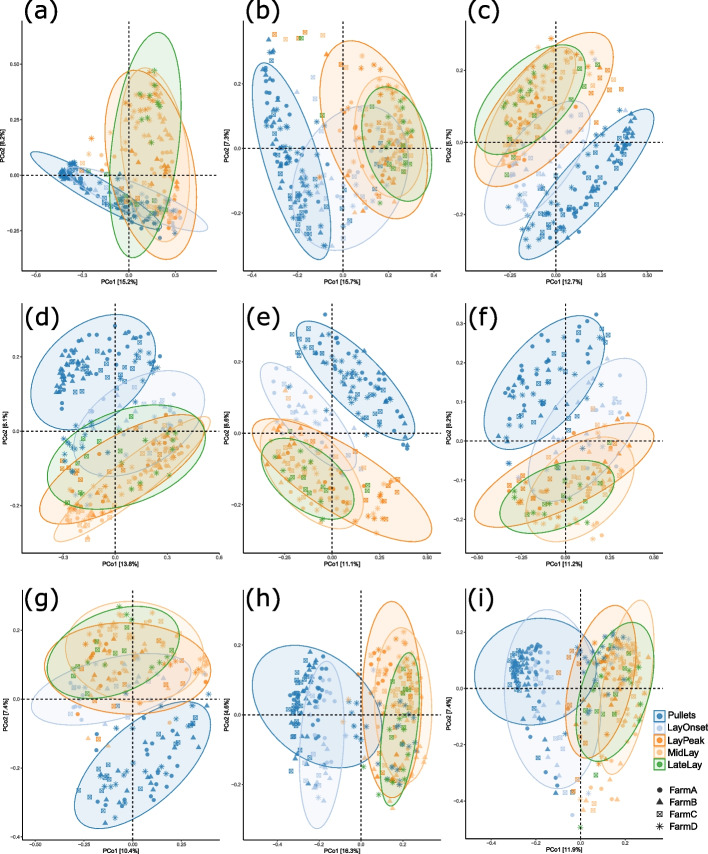
Temporal variation in microbiota population in different gut section. Ordination of individual samples with principal coordinate analysis (PCoA) of Jaccard distance depicting temporal differences in microbiota at different stages of laying lifecycle. This analysis is based on presence or absence of specific microbes. (**a**) Oesophagus. (**b**) Crop. (**c**) Proventriculus. (**d**) Gizzard. (**e**) Duodenum. (**f**) Jejunum. (**g**) Ileum. (**h**) Caecum. (**i**) Colon

**Fig. 6:**
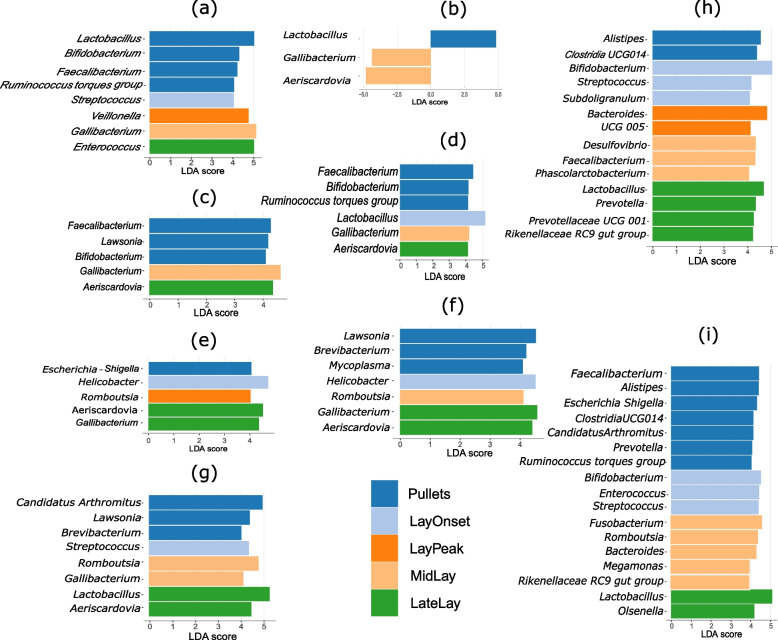
Temporal variation in differential genera in different gut sections. Differential genera identified with the linear discriminant analysis (LDA) effect size (LEfSe) tool with LDA scores of greater than 4 in different stages of laying life cycles are presented. (**a**) Oesophagus. (**b**) Crop. (**c**) Proventriculus. (**d**) Gizzard. (**e**) Duodenum. (**f**) Jejunum. (**g**) Ileum. (**h**) Caecum. (**i**) Colon

**Fig. 7:**
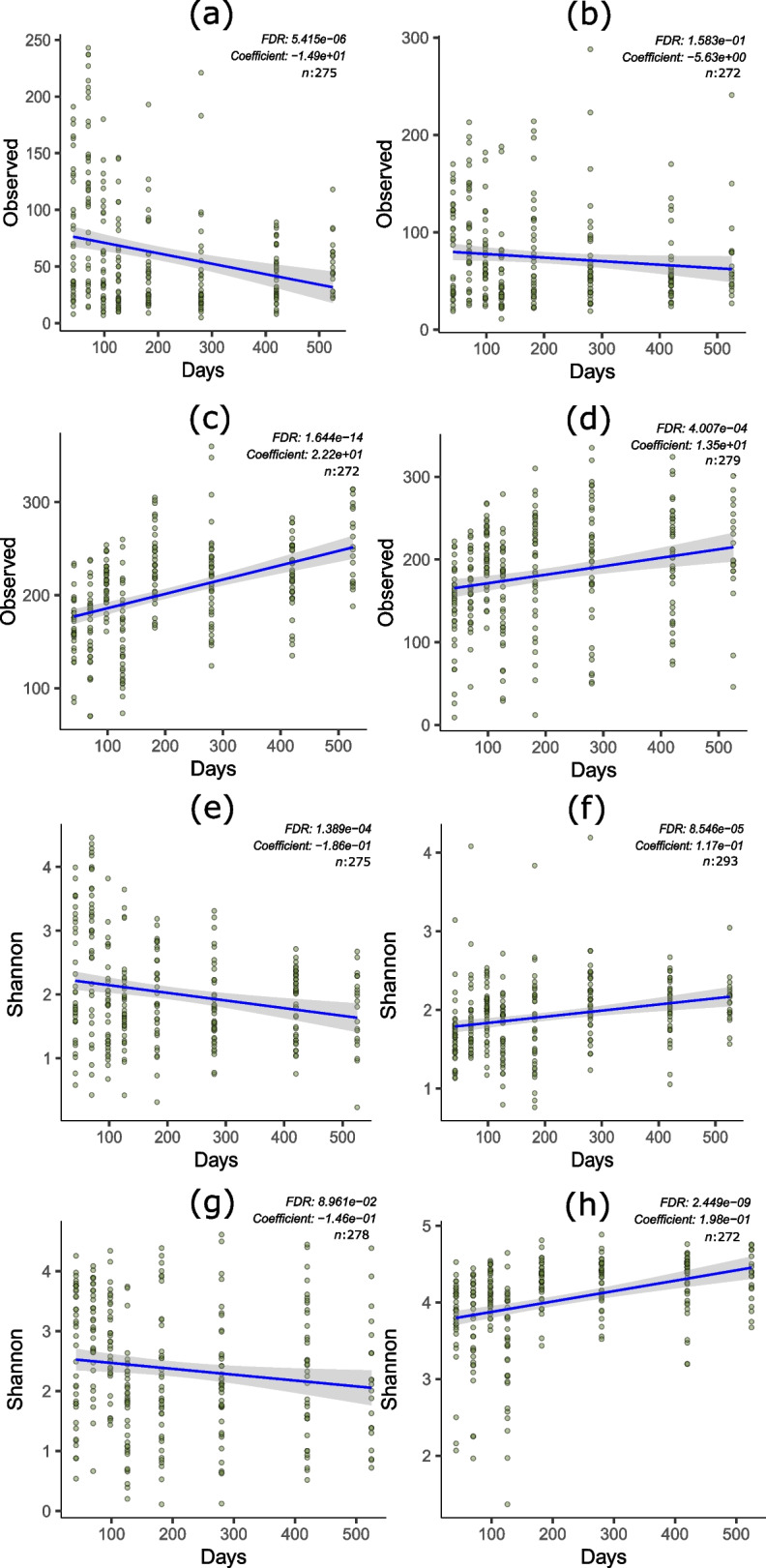
Temporal dynamics of microbiota richness and alpha diversity. Association between microbiota richness measured with Observed taxa and age analysed with general linear models. (**a**) Oesophagus. (**b**) Proventriculus. (**c**) Cecum. (**d**) Colon. Association between microbial alpha diversity measured with Shannon index and age analysed with general linear models. (**e**) Oesophagus. (**f**) Crop. (**g**) Gizzard. (**h**) Cecum. Figures also show false discovery rate (FDR) adjusted *P*-value, coefficient of relationship and number of samples

Figures 5, 6 and 7 depict the temporal variation of microbiota profiles in different sections of the digestive system. It is evident that pullets had the most strikingly different microbiota population compared to other stages of the laying phase in all sections of the gastrointestinal tract (Fig. 5, Fig. S3). As expected, the microbiota profile at lay onset had overlapping microbiota profiles in the PCoA plots both with pullets and later stages of laying phase (Fig. 5, Fig. S3). Microbiota of peak laying stage, mid lay and late lay was mostly overlapped in all sections of the GIT. These temporal fluctuations observed were consistent across all four farms, demonstrating their repeatability (Fig. 5, Fig. S3). The difference in microbiota profile between laying phases is more prominent when compared with Jaccard distance as compared to Bray–Curtis distance (Fig. 5, Fig. S3).

The significance of difference in microbiota composition among different stages of laying cycle was tested with permutational multivariate analysis of variance (PERMANOVA). The “farm” was set as blocking factor in PERMANOVA model to control for any farm-specific differences that may exist and to ensure that any observed differences related to the temporal stage were not confounded with the variation among farms. The PERMANOVA results also confirmed that there were significant differences (*P* < 0.01) in microbiota composition in each gut section both at OTU and phylum level.

Figure 6 presents the microbial genera that were most characteristic of different sections of the GIT in different stages of the lifecycle. To show the bacterial genera with the most significant differential abundance, only genera with an LDA score of more than 4 are presented. In the oesophagus, pullets had a significantly higher abundance of *Lactobacillus, Bifidibacterium, Faecalubacterium* and *Ruminococcus torques group* while *Streptococcus* was the characterising genus at the lay onset (Fig. 6a). The genus *Veillonella* was enriched at peak laying stage compared to other stages while genera *Gallibacterium* and *Enterococcus* were enriched during mid lay and late lay respectively (Fig. 6a).

In the crop, *Lactobacillus* was the characterising genus in pullets, while *Gallibacterium* and *Aeriscardovia* characterised the mid-lay stage (Fig. 6b). Other laying phases had no bacterial genera with differential abundance in the crop. *Fecalibacterium, Lawsonia* and *Bifidobacterium* were the characterising bacterial genera in the proventriculus in pullets (Fig. 6c). *Gallibacterium,* and *Aeriscardovia* were the differential genera in mid-lay and late-lay phase, respectively in the proventriculus. The gizzard of pullets had *Fecalibacterium, Bifidobacterium,* and *Ruminococcus torques group* as characteristic genera, while *Lactobacillus*, *Gallibacterium* and *Aeriscardovia* were characteristic to lay onset, mid lay, and late lay respectively in the gizzard (Fig. 6d).

*Escherichia-Shigella, Helicobacter,* and *Romboutsia* were characteristic of pullets, lay onset, and peak lay stage, respectively in the duodenum (Fig. 6e). *Aeriscardovia* and *Gallibacterium* were characterising genera in the duodenum in late lay stage. In the jejunum, *Lawsonia, Brevibacterium,* and *Mycoplasma* were differential genera for pullets, while *Helicobacter* and *Romboutsia* were characteristic of lay onset and mid-lay stages, respectively (Fig. 6f). *Gallibacterium* and *Aeriscardovia* were enriched in the jejunum in late lay stage compared to other stages. Similarly, *Candidatus Arthromitus* (renamed as *Candidatus Savagella*)*, Lawsonia,* and *Brevibacterium* were characteristic of pullets in the ileum, while *Streptococcus* was enriched on lay onset (Fig. 6i). During the mid-lay phase, *Romboutsia* and *Gallibacterium* were enriched in the ileum followed by enrichment of *Lactobacillus* and *Aeriscardovia* during the late lay phase.

*Alistipes,* and *Clostridia UCG014* in pullets; *Bifidobacterium*, *Streptococcus*, and *Subdoligranulum* on lay onset; and *Bacteroides* and *UCG 005* during the peak of lay were characteristic genera in the cecum. During mid-lay, *Desulfovibrio*, *Faecalibacerium*, and *Phascolarctoacterium* were enriched, while *Lactobacillus*, *Prevotella*, *UCG 001*, and *RC9 gut group* were enriched during late lay in the cecum. The characterising genera in the colon in pullets were *Faecalibacerium, Alistipes, Escherichia-Shigella, Clostridia UCG014, Candidatus Arthromitus* (renamed as *Candidatus Savagella*)*, Prevotella,* and *Ruminococcus torques group. Bifidobacterium*, *Enterococcus*, and *Streptococcus* during lay onset; *Fusobacterium*, *Romboutsia*, *Bacteroides*, *Megamonas,* and *RC9 gut group* during mid lay; and *Lactobacillus* and *Olsenella* during late lay period were enriched in the colon.

### Temporal differences in microbiota richness and diversity

The microbial richness and diversity were also changed temporarily in layer chickens. The Observed species for the richness and the Shannon diversity index for the alpha diversity were chosen indicators for richness and diversity. The test of association between the age of the birds and the microbial richness (Observed species) with linear regression models showed that microbial richness decreased with age in the oesophagus and proventriculus. In contrast, the richness increased with age in the cecum and colon (Fig. 7). Similarly, as measured with Shannon index, the microbial diversity decreased with age in the oesophagus and gizzard. In contrast, the diversity increased with age in the crop and caecum (Fig. 7). The richness (as measured with the Observed species) and diversity (as measured with the Shannon index) were not associated with the age in other organs.

### Variation among farms

The difference in microbiota profile among farms was analysed with principal coordinate analysis (PCoA) and PERMANOVA in each organ. The PERMANOVA model used stage of laying phase and farm as the explanatory variables. The analysis showed that farm had a significant effect (*P* < 0.01) on microbiota profile in all gut sections except the ileum and the proventriculus. However, the PCoA plot of Bray–Curtis distance (Fig. S4) shows large overlap among farms, indicating that while there are some differences in microbiota composition, the overall structure of the communities is relatively similar among farms. Despite some differences in microbiota profile among farms, the overall trend of microbiota variation between different laying phases remained similar across all farms (Fig. 5, Fig. S3). This indicates that temporal variation is more prominent than the variation among farms.

Farm specific differential taxa at the genus level were identified with LEfSe (Fig. S5). The number of differential taxa among farms is less than the number of differential taxa between laying phases, underscoring that temporal changes are more significant than differences among farms. Farm A had higher relative abundance of *Galibacterium* in oesophagus and crop; *Mycoplasma* and *Streptococcus* in jejunum; *Alistipes*, *Prevotella*, *Clostridia UCG-014* and *Fecalibacterium* in cecum; and *Prevotella* and *Streptococcus* in colon. In farm B there was higher abundance of *Bifidobacterium* in oesophagus; *Lactobacillus* in crop; *Fecalibacterium*, *Bifidobacterium* and *Olsenella* in proventriculus; *Fecalibacterium* in gizzard; *Bifidobacterium*, *Olsenella* and *Subdoligranulum* in cecum and *Candidatus Arthromitus* and *Subdoligranulum* in colon. Farm C had higher abundance of *Enterococcus* in oesophaugus, crop, jejunum, and ileum; *Streptococcus* and *Aeriscardovia* in ileum; and *Bifidobacterium* and *Fusobacterium* in colon. Farm D had higher abundance of *Aeriscardovia* in crop, proventriculus, gizzard; *Gallibacterium* and *Veillonella* in proventriculus; and *Bacteroides* in cecum and colon. The outbreak of SLD in farm A and the subsequent antibiotic treatment did not result any dramatic and permanent changes in microbiota profile as the microbiota in farm A followed the similar temporal dynamics as in other flocks (Fig. 5, Fig. S3). However, any immediate short-term effect of the disease outbreak and antibiotic treatment was not studied.

**Fig. 8:**
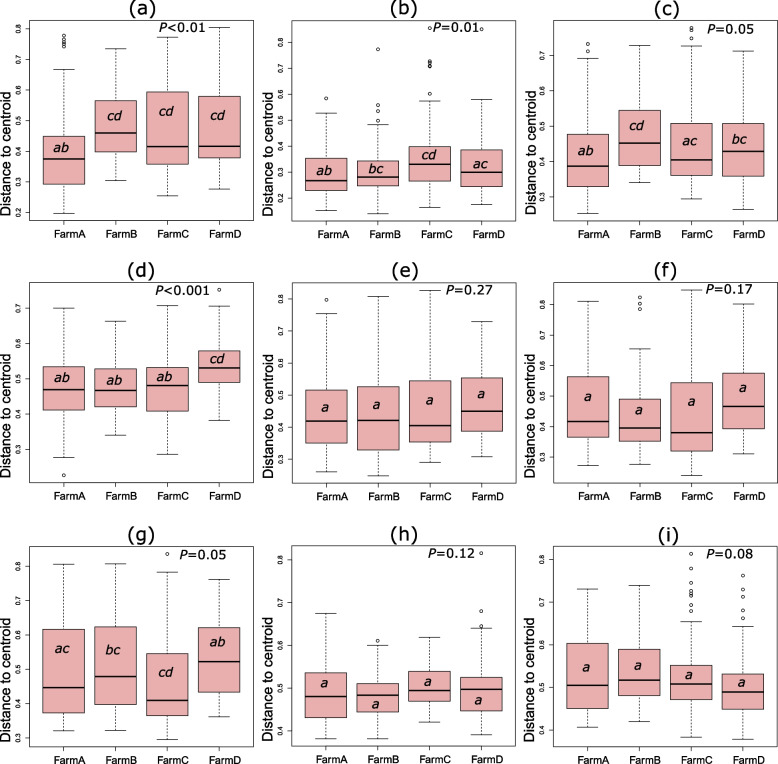
The homogeneity of dispersion among farms. The Bray–Curtis distance of each sample to the centroid of the group was analysed to study the dispersion. The *P* values show the significance of difference among farms analysed with ANOVA. Farms sharing at least one common letter (a or b or c or d) had similar dispersion of samples (Tukey's HSD *P* > 0.05). (**a**) Oesophagus. (**b**) Crop. (**c**) Proventriculus. (**d**) Gizzard. (**e**) Duodenum. (**f**) Jejunum. (**g**) Ileum. (**h**) Caecum. (**i**) Colon

The sample-to-sample variability of microbiota composition among farms or the homogeneity of dispersion was also analysed. The difference in the dispersion of samples among farms was more significant in upper gut (oesophagus, crop, proventriculus and gizzard) than in small intestine and lower gut (Fig. 8). Pairwise comparison of sample-to-sample dispersion with Tukey HSD test between farms in all organs showed that there was no clear pattern of the effects of farm (or production system) (Fig. 8).

## Discussion

The present study aimed to investigate the microbiota composition within all sections of the gastrointestinal tract of healthy layer chickens. The results revealed the dominant bacterial taxa present in the chicken digestive system, and their temporal variation. The most abundant phyla identified in this study were Firmicutes, Actinobacteriota, Proteobacteria, and Bacteroidota; consistent with previous reports on intestinal microbiota of layers [24, 25].

Figure 5 demonstrated that the microbiota structure in pullets and at lay-onset was strikingly different from that in later stages. The overall microbiota structure in all organs changed after the onset of lay and the microbiota at peak lay, mid-lay and late-lay were similar. This indicates that the microbiota stabilised once peak lay was reached and remained relatively unchanged later in life. The changing microbiota going from rearing to the onset of lay and then to peak lay is probably driven by the combined effects of the transfer of pullets from rearing to the production facility; with the concomitant physical housing changes, change in diet, gut maturation, and laying-related physiological changes. This is consistent with previous reports from other studies [26, 27]. Also, the more prominent differences seen, when using Jaccard distance as compared to Bray–Curtis distance, between laying phases indicated the presence of distinct taxa in each phase, but the difference is more subtle when abundance is also considered. This implies that the differences between the laying phases is characterised by less dominant microbial taxa while each phase supports stable abundance of common and dominant taxa. Therefore, any differences in microbial function between different phases could probably be attributable to less dominant taxa.

LEfSe analysis demonstrated that the proventriculus and gizzard had overlapping characterising genera, and jejunum and ileum had overlapping characterising genera in different stages, possibly due to their anatomical proximity. The oesophagus had the highest proportion of Proteobacteria, a bacterial phylum containing a wide variety of pathogens, such as *Salmonella*, *Escherichia-Shigella*, *Gallibacterium*, etc. Genus level data showed that the oesophagus contained a high proportion of *Gallibacterium* (> 25%) (Fig. 1). The genus *Gallibacterium* consists of poultry pathogens, for example, *Gallibacterium anatis* an important opportunistic pathogen in poultry that causes diseases of the respiratory and reproductive systems [28]. Although we were unable to identify the bacteria at the species level with the methods used in this study, it may be worthwhile to conduct epidemiological studies to ascertain the possibility of transmission of *Gallibacterium* from the gastrointestinal tract to the respiratory and reproductive systems. Investigation of temporal development showed that *Gallibacterium* was enriched mostly after peak production (mid-lay and late-lay). *Gallibacterium* was enriched during mid-lay in the oesophagus, crop, proventriculus, gizzard and ileum and enriched during late-lay in the duodenum and jejunum. It is important to note that the flocks sampled during this study did not show any respiratory signs or drop in egg production.

Previous study has shown that the microbiota present in poultry feed may contain up to 39% of Proteobacteria [9]. As the oesophagus is the most anterior section of the digestive system that was sampled, the proportion of Proteobacteria in the oesophagus most probably represents the Proteobacteria in the feed or environmental dust on feed. This warrants a closer investigation of whether feed or the shed environment may make significant contributions to the introduction bacterial pathogens to layers.

The crop had the highest relative abundance of *Lactobacillus* and this is one of the characteristic genera of this organ, as identified by LEfSe. A high abundance of *Lactobacillus* in the crop may have some biological significance as lactobacilli in the crop can inhibit pathogens, for example, *Escherichia coli* introduced through feed or other external environments, by the bacteriostatic and bactericidal activity of some *Lactobacillus* strains [29]. High *Lactobacillus* numbers in the crop may also help to initiate digestion. The saliva of chickens has no significant amylolytic activity [30]; therefore, the breakdown of carbohydrates in the mouth is not as significant as it is in humans and other animals. However, *Lactobacillus* isolated from the crop of chickens can produce amylase and break down carbohydrates [31], which may eventually help to further breakdown the feed particles in the gizzard. Commercial poultry production often uses enzymes as feed supplements. These supplemented enzymes function optimally in slightly acidic pH [32]. The acidic environment of the crop due to higher *Lactobacillus* abundance may help in early activation of the supplemented enzymes in the feed. Intriguingly, the results showed that younger birds (pullets) had *Lactobacillus* as a characteristic genus in the upper gut (oesophagus and crop), while older birds (late lay) had *Lactobacillus* as a characteristic genus in the lower gut (ileum, cecum and colon). *Lactobacillus* was enriched in the gizzard at the onset of lay.

*Aeriscardovia* was characteristic of many intestinal sections, mainly in the late laying phase. This genus was enriched in proventriculus, gizzard, duodenum, jejunum, and ileum in older birds. The bacterial genus *Aeriscardovia* belongs to the family Bifidobacteriaceae, and has received limited attention. However, this genus has been frequently reported as a member of chicken gut microbiota in recent times [33–36]. Members of the genus *Aeriscardovia* are metabolically versatile, capable of utilising a wide range of carbon sources and display both acetate and lactate fermentative metabolisms [37]. Further research is required to elucidate the specific functions and interactions of *Aeriscardovia* within the chicken intestinal microbiota and its potential implications for host health and productivity.

The results that indicate associations between the age of birds and microbial richness and diversity in different organs provide insights into the dynamic nature of the chicken intestinal microbiota throughout their lifespan. The observed patterns of microbial richness and diversity changed with age and highlight the influence of host development and maturation on the composition of the microbiota in specific gastrointestinal regions. The microbial richness and diversity tended to decrease with age in the upper gut, remained constant in the small intestine, and increases with age in the lower gut (caecum and colon). Organ specific changes in microbiota colonisation with age has been demonstrated also in broiler chicken in a recent study [38].

Since the upper gut is involved in the early stages of food intake and digestion, changes in dietary composition may have a greater influence on the composition of the microbial community in this part of the digestive system. Therefore, a decrease in microbial diversity and richness in the upper gut over time may at least partially be attributable to the diet. Conversely, the observed increase in microbial abundance in the lower gut with age suggests ongoing colonisation and diversification of the microbiota in these areas, as birds mature. The caecum and colon are sites of microbial fermentation and play an important role in microbial digestion of fibre in the diet. The age-related increase in microbial richness and diversity in these regions is likely due to the development of specialised microbial communities adapted to the microbial digestion of the fibre in the diet.

It is important to note that age-related changes in microbial abundance and diversity were not consistently observed in all organs. This suggests that factors other than age, such as organ-specific functional and anatomical variations, may also play an important role in the maturation of microbiota in specific gut regions.

## Conclusions

Addressing a gap in previous research focused mainly on one or two gut sections within controlled environments, this study explored the microbiota across all major gut sections and tracked their dynamics from rearing to the end of the production cycle in commercially raised layer chickens. This study provides a comprehensive understanding of spatial and temporal dynamics of microbiota in layer chickens. The finding of distinct structures of microbiota in the rearing and production phase together with the observed fluctuations in putative opportunistic pathogens help to develop more holistic and targeted strategies to optimise gut health and overall productivity in commercial poultry production.

### Supplementary Information

**Additional file 1: Fig. S1.** Top 10 phylum in different sections of the gut in commercially raised layer chickens showing variations among individual birds. **Fig. S2.** Top 20 genera in different sections of the gut in commercially raised layer chickens showing variations among individual birds. **Fig. S3.** Temporal variation in microbiota population in different gut sections. **Fig. S4.** Differences in microbiota population among farms. **Fig. S5.** Flock specific differential genera. **Table S1.** Differences in alpha diversity.

## Data Availability

The datasets generated and/or analysed during the current study are available in the the National Center for Biotechnology Information (NCBI) SRA repository with accession number PRJNA895927, https://www.ncbi.nlm.nih.gov/bioproject/PRJNA895927.

## Abbreviations

ASV: Amplicon sequence variant
FDR: False discovery rate
LEfSe: Linear discriminant analysis effect size
OTU: Operational taxonomic unit
PCoA: Principal coordinate analysis
PERMANOVA: Permutational multivariate analysis of variance
QIIME2: Quantitative insights into microbial ecology 2
SCFA: Short-chain fatty acid
SLD: Spotty liver disease
