# Supplementary material for: Layer chicken microbiota: a comprehensive analysis of spatial and temporal dynamics across all major gut sections
Source: J Anim Sci Biotechnol. 2024 Feb 5;15:20. doi: 10.1186/s40104-023-00979-1 (PMC10840231; doi:10.1186/s40104-023-00979-1)
Supplement: Supplementary file 1 — Additional file 1: Fig. S1. Top 10 phylum in different sections of the gut in commercially raised layer chickens showing variations among individual birds. Fig. S2. Top 20 genera in different sections of the gut in commercially raised layer chickens showing variations among individual birds. Fig. S3. Temporal variation in microbiota population in different gut sections. Fig. S4. Differences in microbiota population among farms. Fig. S5. Flock specific differential genera. Table S1. Differences in alpha diversity. [file 40104_2023_979_MOESM1_ESM.docx]

**Layer chicken microbiota: a comprehensive analysis of spatial and temporal dynamics across all major gut sections**

**Supplementary materials**

Yadav Sharma Bajagai^1*^, Thi Thu Hao Van^2^, Nitish Joat^3^, Kapil Chousalkar^3^, Robert J. Moore^2^, and Dragana Stanley^1^

^1^Institute for Future Farming Systems, Central Queensland University, Rockhampton, Queensland, 4701, Australia

^2^School of Science, RMIT University, Bundoora, Victoria, 3083, Australia

^3^School of Animal and Veterinary Sciences, The University of Adelaide, Roseworthy, South Australia, 5371, Australia

^*^Corresponding author

**Fig. S1** Top 10 phylum in different sections of the gut in commercially raised layer chickens showing variations among individual birds. Oes = Oesophagus, Pro = Proventriculus, Gizz = Gizzard, Duo = Duodenum, Jej = Jejunum, Ile = Ileum, Cae = Caecum, Col = Colon

**Fig. S2** Top 20 genera in different sections of the gut in commercially raised layer chickens showing variations among individual birds. Oes = Oesophagus, Pro = Proventriculus, Gizz = Gizzard, Duo = Duodenum, Jej = Jejunum, Ile = Ileum, Cae = Caecum, Col = Colon

**Fig. S3** Temporal variation in microbiota population in different gut sections. Ordination of individual samples with Principal Coordinate Analysis (PCoA) of Bray-Curtis distance depicting temporal differences in microbiota at different stages of laying lifecycle. This analysis is based on the presence-absence and abundance of specific microbes. **a)** Oesophagus; **b)** Crop; **c)** Proventriculus; **d)** Gizzard; **e)** Duodenum; **f)** Jejunum; **g)** Ileum; **h)**; Caecum **i)** Colon

**Fig. S4** Differences in microbiota population among farms. Ordination of individual samples with Principal Coordinate Analysis (PCoA) of Bray-Curtis distance showing similarity in microbiota profile in different farms. This analysis is based on the presence-absence and abundance of specific microbes. **a)** Oesophagus; **b)** Crop; **c)** Proventriculus; **d)** Gizzard; **e)** Duodenum; **f)** Jejunum; **g)** Ileum; **h)** Caecum; **i)** Colon

**Fig. S5** Flock specific differential genera. Differential genera were identified with Linear discriminant analysis Effect Size (LEfSe) tool with LDA scores of greater than 4 in different farms are presented. **a)** Oesophagus; **b)** Crop; **c)** Proventriculus; **d)** Gizzard; **e)** Jejunum; **f)** Ileum; **g)** Caecum; **h)** Colon

**Table S1** Differences in alpha diversity. Pairwise comparisons of microbiota richness and diversity by Dunn's Kruskal-Wallis Multiple Comparisons test

| **Measure** | **Group** |  | **Comparison** | **Z** | ***P*.unadj** | ***P*.adj** | **Significance** |
| --- | --- | --- | --- | --- | --- | --- | --- |
| Observed | Cae |  | Cae - Col | 2.176432199 | 0.029523 | 0.265706625 | ns |
| Observed | Cae |  | Cae - Crop | 23.62231212 | 2.27E-123 | 8.19E-122 | *** |
| Observed | Col |  | Col - Crop | 21.56047031 | 4.22E-103 | 1.48E-101 | *** |
| Observed | Cae |  | Cae - Duo | 20.28983405 | 1.58E-91 | 5.38E-90 | *** |
| Observed | Col |  | Col - Duo | 18.32738351 | 5.00E-75 | 1.50E-73 | *** |
| Observed | Duo |  | Crop - Duo | -1.91932105 | 0.054944 | 0.439549725 | ns |
| Observed | Cae |  | Cae - Gizz | 11.79222169 | 4.28E-32 | 1.03E-30 | *** |
| Observed | Col |  | Col - Gizz | 9.679289811 | 3.69E-22 | 8.12E-21 | *** |
| Observed | Gizz |  | Crop - Gizz | -11.7438229 | 7.60E-32 | 1.75E-30 | *** |
| Observed | Gizz |  | Duo - Gizz | -9.121119338 | 7.44E-20 | 1.56E-18 | *** |
| Observed | Cae |  | Cae - Ile | 19.12942624 | 1.44E-81 | 4.45E-80 | *** |
| Observed | Col |  | Col - Ile | 17.19027782 | 3.14E-66 | 8.48E-65 | *** |
| Observed | Ile |  | Crop - Ile | -2.738126006 | 0.006179 | 0.061790396 | ns |
| Observed | Ile |  | Duo - Ile | -0.804079568 | 0.421351 | 1 | ns |
| Observed | Gizz |  | Gizz - Ile | 8.133388757 | 4.17E-16 | 7.51E-15 | *** |
| Observed | Cae |  | Cae - Jej | 19.7424871 | 9.31E-87 | 3.07E-85 | *** |
| Observed | Col |  | Col - Jej | 17.8312675 | 4.04E-71 | 1.17E-69 | *** |
| Observed | Jej |  | Crop - Jej | -1.820230407 | 0.068724 | 0.481067471 | ns |
| Observed | Jej |  | Duo - Jej | 0.039423638 | 0.968553 | 0.968552634 | ns |
| Observed | Gizz |  | Gizz - Jej | 8.895297716 | 5.83E-19 | 1.17E-17 | *** |
| Observed | Ile |  | Ile - Jej | 0.822012234 | 0.41107 | 1 | ns |
| Observed | Cae |  | Cae - Oes | 19.67065918 | 3.85E-86 | 1.23E-84 | *** |
| Observed | Col |  | Col - Oes | 17.61343589 | 1.94E-69 | 5.44E-68 | *** |
| Observed | Oes |  | Crop - Oes | -3.65448887 | 0.000258 | 0.003092338 | ** |
| Observed | Oes |  | Duo - Oes | -1.537461099 | 0.12418 | 0.745082605 | ns |
| Observed | Gizz |  | Gizz - Oes | 7.953386124 | 1.81E-15 | 3.09E-14 | *** |
| Observed | Oes |  | Ile - Oes | -0.673966876 | 0.500332 | 1 | ns |
| Observed | Oes |  | Jej - Oes | -1.533750767 | 0.125091 | 0.625454896 | ns |
| Observed | Cae |  | Cae - Pro | 15.15510415 | 7.01E-52 | 1.82E-50 | *** |
| Observed | Col |  | Col - Pro | 13.07463468 | 4.60E-39 | 1.15E-37 | *** |
| Observed | Pro |  | Crop - Pro | -8.188133948 | 2.65E-16 | 5.04E-15 | *** |
| Observed | Pro |  | Duo - Pro | -5.799686715 | 6.64E-09 | 1.06E-07 | *** |
| Observed | Gizz |  | Gizz - Pro | 3.445322446 | 0.00057 | 0.006274164 | ** |
| Observed | Pro |  | Ile - Pro | -4.870366526 | 1.11E-06 | 1.56E-05 | *** |
| Observed | Pro |  | Jej - Pro | -5.672503698 | 1.41E-08 | 2.11E-07 | *** |
| Observed | Pro |  | Oes - Pro | -4.474053067 | 7.68E-06 | 9.98E-05 | *** |
| Chao1 | Cae |  | Cae - Col | 1.959690103 | 0.050032 | 0.350224159 | ns |
| Chao1 | Cae |  | Cae - Crop | 23.34251671 | 1.64E-120 | 5.91E-119 | *** |
| Chao1 | Col |  | Col - Crop | 21.49962215 | 1.57E-102 | 5.49E-101 | *** |
| Chao1 | Cae |  | Cae - Duo | 20.21464102 | 7.28E-91 | 2.47E-89 | *** |
| Chao1 | Col |  | Col - Duo | 18.45887483 | 4.42E-76 | 1.33E-74 | *** |
| Chao1 | Duo |  | Crop - Duo | -1.728683663 | 0.083866 | 0.503194361 | ns |
| Chao1 | Cae |  | Cae - Gizz | 11.40666688 | 3.87E-30 | 8.91E-29 | *** |
| Chao1 | Col |  | Col - Gizz | 9.509202988 | 1.92E-21 | 4.23E-20 | *** |
| Chao1 | Gizz |  | Crop - Gizz | -11.85518266 | 2.02E-32 | 4.85E-31 | *** |
| Chao1 | Gizz |  | Duo - Gizz | -9.414018091 | 4.78E-21 | 1.00E-19 | *** |
| Chao1 | Cae |  | Cae - Ile | 19.05657813 | 5.79E-81 | 1.85E-79 | *** |
| Chao1 | Col |  | Col - Ile | 17.32080055 | 3.28E-67 | 9.18E-66 | *** |
| Chao1 | Ile |  | Crop - Ile | -2.549464963 | 0.010789 | 0.107888351 | ns |
| Chao1 | Ile |  | Duo - Ile | -0.802978654 | 0.421987 | 0.843974145 | ns |
| Chao1 | Gizz |  | Gizz - Ile | 8.422722225 | 3.68E-17 | 6.99E-16 | *** |
| Chao1 | Cae |  | Cae - Jej | 19.74839582 | 8.28E-87 | 2.73E-85 | *** |
| Chao1 | Col |  | Col - Jej | 18.03825532 | 9.76E-73 | 2.83E-71 | *** |
| Chao1 | Jej |  | Crop - Jej | -1.555130473 | 0.119915 | 0.599574994 | ns |
| Chao1 | Jej |  | Duo - Jej | 0.11533338 | 0.908181 | 0.908180881 | ns |
| Chao1 | Gizz |  | Gizz - Jej | 9.258913523 | 2.07E-20 | 4.13E-19 | *** |
| Chao1 | Ile |  | Ile - Jej | 0.895790552 | 0.370365 | 1 | ns |
| Chao1 | Cae |  | Cae - Oes | 18.94681955 | 4.69E-80 | 1.45E-78 | *** |
| Chao1 | Col |  | Col - Oes | 17.10233071 | 1.43E-65 | 3.85E-64 | *** |
| Chao1 | Oes |  | Crop - Oes | -4.111142578 | 3.94E-05 | 0.000511818 | *** |
| Chao1 | Oes |  | Duo - Oes | -2.15399695 | 0.03124 | 0.249923289 | ns |
| Chao1 | Gizz |  | Gizz - Oes | 7.612209421 | 2.69E-14 | 4.58E-13 | *** |
| Chao1 | Oes |  | Ile - Oes | -1.281770697 | 0.199923 | 0.799692386 | ns |
| Chao1 | Oes |  | Jej - Oes | -2.211431108 | 0.027006 | 0.243053979 | ns |
| Chao1 | Cae |  | Cae - Pro | 14.98301564 | 9.48E-51 | 2.47E-49 | *** |
| Chao1 | Col |  | Col - Pro | 13.11819858 | 2.59E-39 | 6.48E-38 | *** |
| Chao1 | Pro |  | Crop - Pro | -8.083595978 | 6.29E-16 | 1.13E-14 | *** |
| Chao1 | Pro |  | Duo - Pro | -5.889031509 | 3.88E-09 | 6.22E-08 | *** |
| Chao1 | Gizz |  | Gizz - Pro | 3.657852616 | 0.000254 | 0.002797709 | ** |
| Chao1 | Pro |  | Ile - Pro | -4.959432207 | 7.07E-07 | 9.90E-06 | *** |
| Chao1 | Pro |  | Jej - Pro | -5.838179227 | 5.28E-09 | 7.92E-08 | *** |
| Chao1 | Pro |  | Oes - Pro | -3.922773216 | 8.75E-05 | 0.001050426 | ** |
| ACE | Cae |  | Cae - Col | 1.963071562 | 0.049638 | 0.347465015 | ns |
| ACE | Cae |  | Cae - Crop | 23.39161702 | 5.20E-121 | 1.87E-119 | *** |
| ACE | Col |  | Col - Crop | 21.54560049 | 5.82E-103 | 2.04E-101 | *** |
| ACE | Cae |  | Cae - Duo | 20.04843866 | 2.08E-89 | 7.08E-88 | *** |
| ACE | Col |  | Col - Duo | 18.29045731 | 9.86E-75 | 2.96E-73 | *** |
| ACE | Crop |  | Crop - Duo | -1.917932952 | 0.05512 | 0.33071708 | ns |
| ACE | Cae |  | Cae - Gizz | 11.28232143 | 1.60E-29 | 3.69E-28 | *** |
| ACE | Col |  | Col - Gizz | 9.380661412 | 6.56E-21 | 1.44E-19 | *** |
| ACE | Gizz |  | Crop - Gizz | -12.03122097 | 2.44E-33 | 5.84E-32 | *** |
| ACE | Gizz |  | Duo - Gizz | -9.378527255 | 6.69E-21 | 1.40E-19 | *** |
| ACE | Cae |  | Cae - Ile | 19.13304316 | 1.34E-81 | 4.29E-80 | *** |
| ACE | Col |  | Col - Ile | 17.39451452 | 9.08E-68 | 2.54E-66 | *** |
| ACE | Ile |  | Crop - Ile | -2.517856459 | 0.011807 | 0.118071428 | ns |
| ACE | Ile |  | Duo - Ile | -0.594278623 | 0.552326 | 1 | ns |
| ACE | Gizz |  | Gizz - Ile | 8.616475509 | 6.90E-18 | 1.31E-16 | *** |
| ACE | Cae |  | Cae - Jej | 19.64469934 | 6.42E-86 | 2.12E-84 | *** |
| ACE | Col |  | Col - Jej | 17.93085704 | 6.77E-72 | 1.96E-70 | *** |
| ACE | Jej |  | Crop - Jej | -1.705934685 | 0.08802 | 0.440101205 | ns |
| ACE | Jej |  | Duo - Jej | 0.148384763 | 0.882039 | 0.882039124 | ns |
| ACE | Gizz |  | Gizz - Jej | 9.270086745 | 1.86E-20 | 3.72E-19 | *** |
| ACE | Ile |  | Ile - Jej | 0.725883623 | 0.46791 | 1 | ns |
| ACE | Cae |  | Cae - Oes | 18.90890649 | 9.63E-80 | 2.99E-78 | *** |
| ACE | Col |  | Col - Oes | 17.06078541 | 2.91E-65 | 7.85E-64 | *** |
| ACE | Oes |  | Crop - Oes | -4.198997519 | 2.68E-05 | 0.000348529 | *** |
| ACE | Oes |  | Duo - Oes | -2.045089271 | 0.040846 | 0.326768463 | ns |
| ACE | Gizz |  | Gizz - Oes | 7.698778683 | 1.37E-14 | 2.34E-13 | *** |
| ACE | Oes |  | Ile - Oes | -1.394081246 | 0.163293 | 0.653172287 | ns |
| ACE | Oes |  | Jej - Oes | -2.142675135 | 0.032139 | 0.28925274 | ns |
| ACE | Cae |  | Cae - Pro | 14.95309622 | 1.49E-50 | 3.87E-49 | *** |
| ACE | Col |  | Col - Pro | 13.08470825 | 4.03E-39 | 1.01E-37 | *** |
| ACE | Pro |  | Crop - Pro | -8.163166663 | 3.26E-16 | 5.87E-15 | *** |
| ACE | Pro |  | Duo - Pro | -5.768427496 | 8.00E-09 | 1.28E-07 | *** |
| ACE | Gizz |  | Gizz - Pro | 3.752115896 | 0.000175 | 0.001928832 | ** |
| ACE | Pro |  | Ile - Pro | -5.064047677 | 4.10E-07 | 5.75E-06 | *** |
| ACE | Pro |  | Jej - Pro | -5.762259908 | 8.30E-09 | 1.24E-07 | *** |
| ACE | Pro |  | Oes - Pro | -3.914861508 | 9.05E-05 | 0.001085473 | ** |
| Shannon | Cae |  | Cae - Col | 3.54271959 | 0.000396 | 0.004752283 | ** |
| Shannon | Cae |  | Cae - Crop | 20.62706579 | 1.57E-94 | 5.49E-93 | *** |
| Shannon | Col |  | Col - Crop | 17.15377051 | 5.89E-66 | 1.77E-64 | *** |
| Shannon | Cae |  | Cae - Duo | 20.67231057 | 6.15E-95 | 2.21E-93 | *** |
| Shannon | Col |  | Col - Duo | 17.4064447 | 7.37E-68 | 2.29E-66 | *** |
| Shannon | Crop |  | Crop - Duo | 1.328846399 | 0.183899 | 1 | ns |
| Shannon | Cae |  | Cae - Gizz | 15.56491299 | 1.26E-54 | 3.15E-53 | *** |
| Shannon | Col |  | Col - Gizz | 12.10231821 | 1.03E-33 | 2.36E-32 | *** |
| Shannon | Gizz |  | Crop - Gizz | -4.888729645 | 1.01E-06 | 1.62E-05 | *** |
| Shannon | Gizz |  | Duo - Gizz | -5.900026204 | 3.63E-09 | 8.00E-08 | *** |
| Shannon | Cae |  | Cae - Ile | 19.76658998 | 5.77E-87 | 1.91E-85 | *** |
| Shannon | Col |  | Col - Ile | 16.54643949 | 1.70E-61 | 4.76E-60 | *** |
| Shannon | Crop |  | Crop - Ile | 0.721553151 | 0.470569 | 1 | ns |
| Shannon | Ile |  | Duo - Ile | -0.553498071 | 0.579922 | 1 | ns |
| Shannon | Gizz |  | Gizz - Ile | 5.226206428 | 1.73E-07 | 3.29E-06 | *** |
| Shannon | Cae |  | Cae - Jej | 19.78240679 | 4.22E-87 | 1.43E-85 | *** |
| Shannon | Col |  | Col - Jej | 16.60405601 | 6.51E-62 | 1.89E-60 | *** |
| Shannon | Crop |  | Crop - Jej | 0.993995987 | 0.320225 | 1 | ns |
| Shannon | Jej |  | Duo - Jej | -0.279393783 | 0.779943 | 1 | ns |
| Shannon | Gizz |  | Gizz - Jej | 5.435476499 | 5.47E-08 | 1.09E-06 | *** |
| Shannon | Ile |  | Ile - Jej | 0.263587802 | 0.792098 | 0.792097559 | ns |
| Shannon | Cae |  | Cae - Oes | 19.53371569 | 5.68E-85 | 1.82E-83 | *** |
| Shannon | Col |  | Col - Oes | 16.10554379 | 2.33E-58 | 6.30E-57 | *** |
| Shannon | Crop |  | Crop - Oes | -0.790213405 | 0.429403 | 1 | ns |
| Shannon | Oes |  | Duo - Oes | -2.05179884 | 0.040189 | 0.442081377 | ns |
| Shannon | Gizz |  | Gizz - Oes | 4.03255765 | 5.52E-05 | 0.000772423 | *** |
| Shannon | Oes |  | Ile - Oes | -1.441480708 | 0.149449 | 1 | ns |
| Shannon | Oes |  | Jej - Oes | -1.700854716 | 0.08897 | 0.889702717 | ns |
| Shannon | Cae |  | Cae - Pro | 15.75134684 | 6.72E-56 | 1.75E-54 | *** |
| Shannon | Col |  | Col - Pro | 12.30836541 | 8.17E-35 | 1.96E-33 | *** |
| Shannon | Pro |  | Crop - Pro | -4.585665402 | 4.53E-06 | 6.79E-05 | *** |
| Shannon | Pro |  | Duo - Pro | -5.612081741 | 2.00E-08 | 4.20E-07 | *** |
| Shannon | Gizz |  | Gizz - Pro | 0.272117242 | 0.785532 | 1 | ns |
| Shannon | Pro |  | Ile - Pro | -4.946540379 | 7.55E-07 | 1.28E-05 | *** |
| Shannon | Pro |  | Jej - Pro | -5.158872275 | 2.48E-07 | 4.47E-06 | *** |
| Shannon | Pro |  | Oes - Pro | -3.739234085 | 0.000185 | 0.002399563 | ** |
| Simpson | Cae |  | Cae - Col | 4.049221 | 5.14E-05 | 0.00097638 | *** |
| Simpson | Cae |  | Cae - Crop | 18.27069349 | 1.42E-74 | 4.67E-73 | *** |
| Simpson | Col |  | Col - Crop | 14.26596729 | 3.57E-46 | 9.27E-45 | *** |
| Simpson | Cae |  | Cae - Duo | 19.71162738 | 1.71E-86 | 6.17E-85 | *** |
| Simpson | Col |  | Col - Duo | 15.9561911 | 2.58E-57 | 7.74E-56 | *** |
| Simpson | Crop |  | Crop - Duo | 2.601426817 | 0.009284 | 0.102120558 | ns |
| Simpson | Cae |  | Cae - Gizz | 16.58972041 | 8.27E-62 | 2.56E-60 | *** |
| Simpson | Col |  | Col - Gizz | 12.62439427 | 1.55E-36 | 3.72E-35 | *** |
| Simpson | Gizz |  | Crop - Gizz | -1.475168387 | 0.140167 | 0.981170949 | ns |
| Simpson | Gizz |  | Duo - Gizz | -3.95518522 | 7.65E-05 | 0.001376557 | ** |
| Simpson | Cae |  | Cae - Ile | 19.25058502 | 1.40E-82 | 4.89E-81 | *** |
| Simpson | Col |  | Col - Ile | 15.55133771 | 1.56E-54 | 4.52E-53 | *** |
| Simpson | Crop |  | Crop - Ile | 2.40950228 | 0.015974 | 0.159742963 | ns |
| Simpson | Ile |  | Duo - Ile | -0.140942856 | 0.887915 | 1 | ns |
| Simpson | Gizz |  | Gizz - Ile | 3.744106179 | 0.000181 | 0.003077631 | ** |
| Simpson | Cae |  | Cae - Jej | 18.99239877 | 1.97E-80 | 6.70E-79 | *** |
| Simpson | Col |  | Col - Jej | 15.33991847 | 4.14E-53 | 1.12E-51 | *** |
| Simpson | Crop |  | Crop - Jej | 2.37355859 | 0.017618 | 0.15855838 | ns |
| Simpson | Jej |  | Duo - Jej | -0.142025118 | 0.88706 | 1 | ns |
| Simpson | Gizz |  | Gizz - Jej | 3.691054161 | 0.000223 | 0.003573225 | ** |
| Simpson | Jej |  | Ile - Jej | -0.002771178 | 0.997789 | 0.997788923 | ns |
| Simpson | Cae |  | Cae - Oes | 17.66886825 | 7.28E-70 | 2.33E-68 | *** |
| Simpson | Col |  | Col - Oes | 13.72091653 | 7.61E-43 | 1.90E-41 | *** |
| Simpson | Crop |  | Crop - Oes | -0.326528673 | 0.744024 | 1 | ns |
| Simpson | Oes |  | Duo - Oes | -2.87131596 | 0.004088 | 0.061315009 | ns |
| Simpson | Gizz |  | Gizz - Oes | 1.129865238 | 0.258533 | 1 | ns |
| Simpson | Oes |  | Ile - Oes | -2.678262014 | 0.007401 | 0.103607423 | ns |
| Simpson | Oes |  | Jej - Oes | -2.639650365 | 0.008299 | 0.107889078 | ns |
| Simpson | Cae |  | Cae - Pro | 15.36108022 | 2.99E-53 | 8.36E-52 | *** |
| Simpson | Col |  | Col - Pro | 11.40912619 | 3.76E-30 | 8.66E-29 | *** |
| Simpson | Pro |  | Crop - Pro | -2.626746297 | 0.008621 | 0.103446697 | ns |
| Simpson | Pro |  | Duo - Pro | -5.024541543 | 5.05E-07 | 1.11E-05 | *** |
| Simpson | Pro |  | Gizz - Pro | -1.145079754 | 0.252176 | 1 | ns |
| Simpson | Pro |  | Ile - Pro | -4.797727557 | 1.60E-06 | 3.37E-05 | *** |
| Simpson | Pro |  | Jej - Pro | -4.7311874 | 2.23E-06 | 4.46E-05 | *** |
| Simpson | Pro |  | Oes - Pro | -2.265722009 | 0.023468 | 0.187747273 | ns |
| InvSimpson | Cae |  | Cae - Col | 4.049284601 | 5.14E-05 | 0.000976114 | *** |
| InvSimpson | Cae |  | Cae - Crop | 18.27072607 | 1.42E-74 | 4.67E-73 | *** |
| InvSimpson | Col |  | Col - Crop | 14.26593531 | 3.57E-46 | 9.28E-45 | *** |
| InvSimpson | Cae |  | Cae - Duo | 19.71165798 | 1.71E-86 | 6.17E-85 | *** |
| InvSimpson | Col |  | Col - Duo | 15.9561611 | 2.58E-57 | 7.74E-56 | *** |
| InvSimpson | Crop |  | Crop - Duo | 2.601426816 | 0.009284 | 0.102120558 | ns |
| InvSimpson | Cae |  | Cae - Gizz | 16.58975258 | 8.27E-62 | 2.56E-60 | *** |
| InvSimpson | Col |  | Col - Gizz | 12.6243627 | 1.55E-36 | 3.72E-35 | *** |
| InvSimpson | Gizz |  | Crop - Gizz | -1.475168386 | 0.140167 | 0.981170949 | ns |
| InvSimpson | Gizz |  | Duo - Gizz | -3.955185219 | 7.65E-05 | 0.001376557 | ** |
| InvSimpson | Cae |  | Cae - Ile | 19.25061512 | 1.39E-82 | 4.88E-81 | *** |
| InvSimpson | Col |  | Col - Ile | 15.55130819 | 1.56E-54 | 4.52E-53 | *** |
| InvSimpson | Crop |  | Crop - Ile | 2.409502279 | 0.015974 | 0.159742963 | ns |
| InvSimpson | Ile |  | Duo - Ile | -0.140942855 | 0.887915 | 1 | ns |
| InvSimpson | Gizz |  | Gizz - Ile | 3.744106178 | 0.000181 | 0.003077631 | ** |
| InvSimpson | Cae |  | Cae - Jej | 18.99242848 | 1.97E-80 | 6.70E-79 | *** |
| InvSimpson | Col |  | Col - Jej | 15.33988935 | 4.14E-53 | 1.12E-51 | *** |
| InvSimpson | Crop |  | Crop - Jej | 2.373558589 | 0.017618 | 0.15855838 | ns |
| InvSimpson | Jej |  | Duo - Jej | -0.142025118 | 0.88706 | 1 | ns |
| InvSimpson | Gizz |  | Gizz - Jej | 3.69105416 | 0.000223 | 0.003573225 | ** |
| InvSimpson | Jej |  | Ile - Jej | -0.002771178 | 0.997789 | 0.997788923 | ns |
| InvSimpson | Cae |  | Cae - Oes | 17.66890033 | 7.28E-70 | 2.33E-68 | *** |
| InvSimpson | Col |  | Col - Oes | 13.72088504 | 7.61E-43 | 1.90E-41 | *** |
| InvSimpson | Crop |  | Crop - Oes | -0.326528673 | 0.744024 | 1 | ns |
| InvSimpson | Oes |  | Duo - Oes | -2.87131596 | 0.004088 | 0.061315009 | ns |
| InvSimpson | Gizz |  | Gizz - Oes | 1.129865238 | 0.258533 | 1 | ns |
| InvSimpson | Oes |  | Ile - Oes | -2.678262013 | 0.007401 | 0.103607423 | ns |
| InvSimpson | Oes |  | Jej - Oes | -2.639650364 | 0.008299 | 0.107889078 | ns |
| InvSimpson | Cae |  | Cae - Pro | 15.36111222 | 2.98E-53 | 8.36E-52 | *** |
| InvSimpson | Col |  | Col - Pro | 11.40909479 | 3.77E-30 | 8.66E-29 | *** |
| InvSimpson | Pro |  | Crop - Pro | -2.626746296 | 0.008621 | 0.103446697 | ns |
| InvSimpson | Pro |  | Duo - Pro | -5.024541542 | 5.05E-07 | 1.11E-05 | *** |
| InvSimpson | Pro |  | Gizz - Pro | -1.145079754 | 0.252176 | 1 | ns |
| InvSimpson | Pro |  | Ile - Pro | -4.797727556 | 1.60E-06 | 3.37E-05 | *** |
| InvSimpson | Pro |  | Jej - Pro | -4.731187399 | 2.23E-06 | 4.46E-05 | *** |
| InvSimpson | Pro |  | Oes - Pro | -2.265722009 | 0.023468 | 0.187747273 | ns |
| Fisher | Cae |  | Cae - Col | 2.176432199 | 0.029523 | 0.265706625 | ns |
| Fisher | Cae |  | Cae - Crop | 23.62231212 | 2.27E-123 | 8.19E-122 | *** |
| Fisher | Col |  | Col - Crop | 21.56047031 | 4.22E-103 | 1.48E-101 | *** |
| Fisher | Cae |  | Cae - Duo | 20.28983405 | 1.58E-91 | 5.38E-90 | *** |
| Fisher | Col |  | Col - Duo | 18.32738351 | 5.00E-75 | 1.50E-73 | *** |
| Fisher | Duo |  | Crop - Duo | -1.91932105 | 0.054944 | 0.439549725 | ns |
| Fisher | Cae |  | Cae - Gizz | 11.79222169 | 4.28E-32 | 1.03E-30 | *** |
| Fisher | Col |  | Col - Gizz | 9.679289811 | 3.69E-22 | 8.12E-21 | *** |
| Fisher | Gizz |  | Crop - Gizz | -11.7438229 | 7.60E-32 | 1.75E-30 | *** |
| Fisher | Gizz |  | Duo - Gizz | -9.121119338 | 7.44E-20 | 1.56E-18 | *** |
| Fisher | Cae |  | Cae - Ile | 19.12942624 | 1.44E-81 | 4.45E-80 | *** |
| Fisher | Col |  | Col - Ile | 17.19027782 | 3.14E-66 | 8.48E-65 | *** |
| Fisher | Ile |  | Crop - Ile | -2.738126006 | 0.006179 | 0.061790396 | ns |
| Fisher | Ile |  | Duo - Ile | -0.804079568 | 0.421351 | 1 | ns |
| Fisher | Gizz |  | Gizz - Ile | 8.133388757 | 4.17E-16 | 7.51E-15 | *** |
| Fisher | Cae |  | Cae - Jej | 19.7424871 | 9.31E-87 | 3.07E-85 | *** |
| Fisher | Col |  | Col - Jej | 17.8312675 | 4.04E-71 | 1.17E-69 | *** |
| Fisher | Jej |  | Crop - Jej | -1.820230407 | 0.068724 | 0.481067471 | ns |
| Fisher | Jej |  | Duo - Jej | 0.039423638 | 0.968553 | 0.968552634 | ns |
| Fisher | Gizz |  | Gizz - Jej | 8.895297716 | 5.83E-19 | 1.17E-17 | *** |
| Fisher | Ile |  | Ile - Jej | 0.822012234 | 0.41107 | 1 | ns |
| Fisher | Cae |  | Cae - Oes | 19.67065918 | 3.85E-86 | 1.23E-84 | *** |
| Fisher | Col |  | Col - Oes | 17.61343589 | 1.94E-69 | 5.44E-68 | *** |
| Fisher | Oes |  | Crop - Oes | -3.65448887 | 0.000258 | 0.003092338 | ** |
| Fisher | Oes |  | Duo - Oes | -1.537461099 | 0.12418 | 0.745082605 | ns |
| Fisher | Gizz |  | Gizz - Oes | 7.953386124 | 1.81E-15 | 3.09E-14 | *** |
| Fisher | Oes |  | Ile - Oes | -0.673966876 | 0.500332 | 1 | ns |
| Fisher | Oes |  | Jej - Oes | -1.533750767 | 0.125091 | 0.625454896 | ns |
| Fisher | Cae |  | Cae - Pro | 15.15510415 | 7.01E-52 | 1.82E-50 | *** |
| Fisher | Col |  | Col - Pro | 13.07463468 | 4.60E-39 | 1.15E-37 | *** |
| Fisher | Pro |  | Crop - Pro | -8.188133948 | 2.65E-16 | 5.04E-15 | *** |
| Fisher | Pro |  | Duo - Pro | -5.799686715 | 6.64E-09 | 1.06E-07 | *** |
| Fisher | Gizz |  | Gizz - Pro | 3.445322446 | 0.00057 | 0.006274164 | ** |
| Fisher | Pro |  | Ile - Pro | -4.870366526 | 1.11E-06 | 1.56E-05 | *** |
| Fisher | Pro |  | Jej - Pro | -5.672503698 | 1.41E-08 | 2.11E-07 | *** |
| Fisher | Pro |  | Oes - Pro | -4.474053067 | 7.68E-06 | 9.98E-05 | *** |
